# A home calendar and recall method of last menstrual period for estimating gestational age in rural Bangladesh: a validation study

**DOI:** 10.1186/s41043-016-0072-y

**Published:** 2016-10-21

**Authors:** Alison D. Gernand, Rina Rani Paul, Barkat Ullah, Muhammad A. Taher, Frank R. Witter, Lee Wu, Alain B. Labrique, Keith P. West, Parul Christian

**Affiliations:** 1 Department of Nutritional Sciences, The Pennsylvania State University, 110 Chandlee Laboratory, University Park, PA 16802 USA; 2 Department of International Health, Johns Hopkins Bloomberg School of Public Health, 615 N. Wolfe Street, Baltimore, MD 21205 USA; 3 The JiVitA Maternal and Child Health and Nutrition Research Project, Godown Road, Gaibandha, Bangladesh; 4 Centre for Nuclear Medicine & Ultrasound, Rangpur Medical College Hospital Campus, G.P.O. Box No. 16, Rangpur, Bangladesh; 5 Division of Maternal-Fetal Medicine, The Johns Hopkins Hospital, 1800 Orleans St, Baltimore, MD 21287 USA

**Keywords:** Last menstrual period, Ultrasound, Validation, Bangladesh, Gestational age, Crown-rump length

## Abstract

**Background:**

The best method of gestational age assessment is by ultrasound in the first trimester; however, this method is impractical in large field trials in rural areas. Our objective was to assess the validity of gestational age estimated from prospectively collected date of last menstrual period (LMP) using crown-rump length (CRL) measured in early pregnancy by ultrasound.

**Methods:**

As part of a large, cluster-randomized, controlled trial in rural Bangladesh, we collected dates of LMP by recall and as marked on a calendar every 5 weeks in women likely to become pregnant. Among those with a urine-test confirmed pregnancy, a subset with gestational age of <15 weeks (*n* = 353) were enrolled for ultrasound follow-up to measure CRL. We compared interview-assessed LMP with CRL gestational age estimates and classification of preterm, term, and post-term births.

**Results:**

LMP-based gestational age was higher than CRL by a mean (SD) of 2.8 (10.7) days; differences varied by maternal education and preterm birth (*P* < 0.05). Lin’s concordance correlation coefficient was good at ultrasound [0.63 (95 % CI 0.56, 0.69)] and at birth [0.77 (95 % CI 0.73, 0.81)]. Validity of classifying preterm birth was high but post-term was lower, with specificity of 96 and 89 % and sensitivity of 86 and 67 %, respectively. Results were similar by parity.

**Conclusions:**

Prospectively collected LMP provided a valid estimate of gestational age and preterm birth in a rural, low-income setting and may be a suitable alternative to ultrasound in programmatic settings and large field trials.

**Trial registration:**

ClinicalTrials.gov NCT00860470

**Electronic supplementary material:**

The online version of this article (doi:10.1186/s41043-016-0072-y) contains supplementary material, which is available to authorized users.

## Background

Globally, an estimated 14.9 million preterm births occurred in 2010 [1]. The global preterm birth rate was 11.1 % of all live births, yet rates were highly variable, ranging from 5 % in Europe to 18 % in some countries in Africa, perhaps in part related to measurement differences. Aside from determination of preterm birth, accurate gestational dating is important for a variety of public health and clinical objectives, including monitoring fetal growth, and this poses a challenge in low-income and middle-income countries (LMIC) [2]. In many LMIC settings, better assessment methods of gestational age are needed in the context of poor antenatal care services, limited ultrasound use, and high burden of preterm birth. The cheapest, most readily available method used in many settings, clinical and research, is the self-reported first day of the last menstrual period (LMP); however, this method has limitations and varying levels of accuracy among different populations [3]. Key limitations of menstrual dating include uncertainty of LMP date due to recall bias, variations in timing of ovulation, and bleeding not due to menses [3]. An early ultrasound exam-based assessment is better at predicting the date of delivery [3], and it is safe and increasingly popular and available, even in LMIC [4].

Studies comparing LMP to ultrasound dating have been typically reported in high-income countries where the resources exist for screening most women [5–7]; however, some have also been done in LMIC [8, 9]. Crown-rump length (CRL) measured in the first trimester provides the most accurate estimate of date of delivery, as supported by the American College of Obstetricians and Gynecologists [10]. Early ultrasound exams have a lower prediction error than those conducted later and may be of particular importance in settings with high rates of fetal growth restriction [5].

In rural northwestern Bangladesh, we conducted a large cluster-randomized trial of antenatal micronutrient supplementation to examine outcomes of low birth weight, preterm birth, and small-for-gestational age [11]. Because over 90 % of women deliver at home and antenatal care is accessed late in pregnancy, we developed a pregnancy surveillance system to identify pregnancies early in gestation. To do this, we developed a method of LMP ascertainment in all women of reproductive age which involved visits every 5 weeks to their homes to ask about LMP dates and conduct urine-based testing. We have previously employed this method of assessing gestational age and estimating rates of preterm birth, and women in these trials were enrolled at a mean gestational age ranging between 9 and 12 weeks [12, 13]. In the multiple micronutrient supplementation trial, in which the intervention was found to significantly reduce the rate of preterm birth by 15 % [11], we conducted a validation substudy using early pregnancy crown-rump length (CRL) to examine the performance of our LMP method and to examine the validity of gestational age and preterm birth rates estimated by LMP. Our goal was to validate this LMP method for use in research studies in LMIC, as ultrasound measurements are expensive and challenging to conduct in resource-limited, rural areas.

## Methods

The validation study was conducted in a subsample of pregnant women participating in a cluster-randomized, controlled trial of antenatal maternal micronutrient supplementation (*n* = 44,567) [11]. Additional details of this substudy have been published elsewhere [14, 15]. For the parent trial, women received either iron and folic acid (standard of care) or a supplement with 15 vitamins and minerals daily. The parent trial was conducted in a ~435 km^2^ area of rural northwestern Bangladesh. The study area was divided into 596 clusters, each of which had a local female field worker who was responsible for identifying and recruiting pregnancies. At the outset, a registry was created and maintained of all married women of reproductive potential in the study area. A Bangla-language calendar was provided to each home, and women were instructed to mark the date of the beginning of their menses on the calendar (if illiterate, getting another household member’s help). The local female workers visited each woman on their register every 5 weeks, asked for a history of the LMP, and recorded the LMP date (defined as the first day of menses) marked by the woman on the calendar or from the woman’s verbal report. If the calendar was unmarked, the female worker updated it, based on the woman’s recall. If the woman reported no menstruation for the past 30 days, she was offered a urine-based pregnancy test. Women who tested positive and gave informed consent were enrolled into the trial and visited weekly thereafter for supplementation. The date of menses obtained just before a positive pregnancy test was used as the reported LMP to estimate the gestational age of the pregnancy. All parous women had to have reported resumption of menstruation after their previous pregnancy. Subsequently, trained interviewers collected maternal characteristics including demographics, socioeconomic factors, and pregnancy history at baseline. Each pregnancy was followed weekly until its outcome. Birth outcomes (including fetal losses and maternal deaths) were recorded by the female workers. Births were notified and a special team of trained anthropometrists visited the home to weigh and measure the newborn. Weight was measured as close to the time of delivery as possible on a digital Tanita Infant Scale (Model BD-585) to the nearest 0.01 kg.

A biochemical substudy was conducted in ~10 % of the parent trial and half of this area was selected for the current study involving ultrasound measurements. The substudy area was purposefully selected to include a wide socioeconomic distribution, representative of the larger study population. However, the substudy area also had better roads overall and was closer to health care facilities. Women (*n* = 445) in the ultrasound study were enrolled from February 2009 to March 2010 at a median (interquartile range) of 9.6 (7.7, 12.1) weeks gestation. Women were eligible if their LMP indicated <15 weeks gestation because CRL is more accurate early in gestation [5], thus women in the substudy area identified as pregnant at ≥15 weeks gestation by LMP were not eligible for the validation study. An additional exclusion criterion was having a multiple pregnancy identified by ultrasound scan. Within a week of consent, women were scheduled to visit a nearby field office, where a private room was outfitted to allow ultrasound measurements to be conducted. Transabdominal ultrasound scans were performed with a portable SonoSite Titan device (Bothell, WA). A local ultrasound expert (MAT) trained two technicians in CRL measurement with 17 training days in two local ultrasound/prenatal clinics and in study field offices over a span of 4 months. A staff physician (RRP) trained in prenatal ultrasound oversaw the technicians and also performed scans. No sex determination was done, and we specifically avoided this issue by conducting early scans. With a full bladder, the woman lay on her back with the abdomen exposed. CRL was measured with the fetus in a natural/neutral posture at 90^o^ to the angle of insonation (horizontal), and calipers were placed at the outer edge of the skin over the head and rump. CRL was measured three times to the nearest 0.01 cm and the longest measurement recorded if gestation was 6–14 weeks using the Hadlock reference [16]. If CRL indicated a gestational age of <6 weeks, the woman was scheduled to return in 1–2 weeks. For quality control, one quarter of measurements were repeated by a second technician/physician. For external quality control, 20 % of all final images from across the study period were reviewed by an obstetrician (FRW) at the Johns Hopkins Hospital. All but two images were considered to be of acceptable quality. One of the unacceptable images was excluded, and the other had an acceptable scan of three images saved that was used instead.

We used the WHO definitions to classify preterm (<259 days), term (259-293 days), and post-term (>293) births. We dichotomized maternal characteristics at enrollment: parity (0 and ≥1), education (≤6 and >6 years), age (≤ 20 and >20 years), and body mass index (BMI; <18.5 and ≥18.5 kg/m^2^). We classified small-for-gestational age (SGA) as birth weight <10th percentile of the new Intergrowth 21st standard [17]. Birth weight measured within 72 h and CRL-based gestational age were used for SGA classification.

Of the 445 women enrolled, four declined the ultrasound scan, 19 had a spontaneous miscarriage, and 16 reported induced abortion before the scan. Scans were performed on 406 women and of these, 19 detected no viable fetus, 25 had a CRL out of range (>15 weeks gestation), and three women had twins, all of whom were considered ineligible and excluded in the analysis. Thus, CRL was measured in 359 singleton pregnancies. Six women were ascertained as pregnant (by ultrasound) but had missing or implausible LMP and were excluded from the analysis. Thus, our analytic sample was 353 singleton pregnancies with both LMP and CRL estimates of gestational age. There were 13 miscarriages or terminations of pregnancy and 8 stillbirths among this final sample, leaving 332 pregnancies in the subset with live births.

### Statistical analysis

We compared maternal, household, and infant characteristics between the women in the substudy and the parent trial using *t* tests for continuous variables and chi-squared tests for categorical variables. The difference in gestational age at birth was calculated as LMP minus the CRL estimate. The difference was approximately normally distributed by visual examination with a Kernel density plot, and there were no extreme statistical outliers, allowing for parametric testing. Differences in mean gestational age were tested using a *t* test overall and stratified by maternal and infant characteristics (term status, parity, education, age, BMI, infant sex, and SGA). Validity of LMP-based gestational age at birth was tested against the CRL-based estimate as the gold standard. First, we calculated Pearson’s correlation coefficient and the bias correction factor, which can be considered measures of precision and accuracy, respectively, in validation analysis [18]. Then, we assessed agreement of the two continuous estimates of gestational age using Lin’s concordance correlation coefficient (CCC, combines accuracy and precision measures) [18]. Finally, sensitivity, specificity, positive predictive value (PPV), and kappa coefficients were calculated for rates of preterm and post-term births. We then used the Intergrowth 21st CRL equation for estimating gestational age (for CRL >15 and <95 mm; *n* = 333) and repeated the validity tests [19]. We calculated the mean difference for LMP-CRL across categories of maternal and infant characteristics (years of school, age, BMI, infant sex, term category, and SGA) and used multiple linear regression with all characteristics as predictors to estimate the difference of the LMP-CRL difference by each characteristic. Stata 13 (StataCorp LP, College Station, TX, USA) was used for analysis.

## Results

Among the women included in this analysis (*n* = 353), 40 % were underweight (BMI <18.5 kg/m^2^) and 38 % were nulliparous at study enrollment. Ownership of household items was low and rates of SGA were high (Table 1). Compared to the parent trial, which included singleton live births, most characteristics did not differ in the substudy. However, the rate of preterm birth was lower (9.9 vs. 19.6 %) and the mean birth weight higher (2.62 vs. 2.55 kg), in addition to women’s height, education, and literacy being higher in the substudy vs. the parent trial (Table 1). Percentage of homes with electricity was 7.1 % lower in the substudy compared to the parent study.

**Table 1:** Characteristics of mothers, infants, and their households in the study sample compared with singletons in the parent trial in rural Bangladesh, 2008–2009

	Parent trial (*n* = 26,476)	Substudy (*n* = 353)	*P*
	Mean ± SD or % (*n*)		
Maternal			
Age, years	22.8 ± 5.5	23.1 ± 5.1	0.18
Height, cm	149.6 ± 5.2	149.1 ± 5.2	0.04
BMI, kg/m^2^	19.3 ± 2.4	19.3 ± 2.4	0.61
BMI <18.5	39.4 (10,369)	40.2 (142)	0.74
MUAC, cm	23.5 ± 2.2	23.7 ± 2.2	0.11
Parity	1.09 ± 1.22	1.05 ± 1.21	0.54
Nulliparous	38.9 (10,297)	38.0 (134)	0.71
Can read or write a Bangla letter	63.1 (16,688)	71.4 (252)	<0.01
Completed >6 years of school	40.9 (10,799)	48.7 (172)	<0.01
Household			
Has electricity	21.8 (5757)	14.7 (52)	<0.01
Owns a mobile phone	37.9 (10,019)	35.1 (124)	0.29
Owns a TV	17.4 (4610)	14.5 (51)	0.14
Infant			
Male	51.2 (13,033)	52.6 (170)	0.60
Birth weight, kg^a^	2.55 ± 0.41	2.62 ± 0.40	0.01
Low birth weight (<2.5 kg)^a^	43.4 (8663)	38.9 (108)	0.13
Small-for-gestational age (Alexander)^b,c^	63.5 (12,694)	68.4 (190)	0.10
Small-for-gestational age (Intergrowth) ^b,c^	51.8 (9376)	54.0 (141)	0.47
Preterm (<37 weeks gestation)^b^	19.6 (4991)	9.9 (32)	<0.01

^a^ *n* = 19,982 (parent trial) and *n* = 278 (substudy) for birth weight and LBW including all infants with weight taken ≤72 h from delivery

^b^Gestational age estimated by date of the last menstrual period to allow comparison between parent trial (without ultrasound) and substudy: preterm *n* = 25,474 (parent), *n* = 323 (substudy)

^c^SGA is <10th percentile of birth weight for gestational age and infant sex by the Alexander et al. reference [25] or the international intergrowth standards [17]. SGA: *n* = 18,119 (parent trial), *n* = 261 (substudy) including all infants with weight taken ≤72 h from delivery

**Fig. 1:**
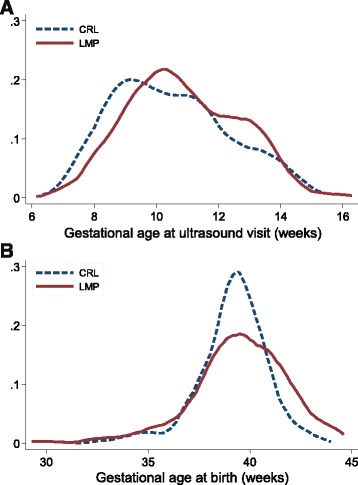
Distribution of gestational age estimations by crown-rump length (CRL) and the first day of the last menstrual period (LMP) at ultrasound visit (*n* = 353, **a**) and live birth (*n* = 332, **b**). Figure excludes three women with LMP-based gestational age <30 weeks

Mean (SD) gestational age at the ultrasound visit was 76.1 (12.5) days [10.9 (1.8) weeks] by LMP and 73.3 (12.9) days [10.5 (1.8) weeks] by CRL (*p* < 0.001), with a mean (SD) difference of 2.8 (10.8) days (Table 2). Gestational age at birth for live births was 276.2 (18.1) days [39.5 (2.6) weeks] by LMP and 273.4 (14.2) days [39.1 (2.0) weeks] by CRL estimation (p < 0.001). The sample size was slightly less at birth (*n* = 332) due to pregnancy loss, but the mean (SD) difference in LMP- and CRL-based gestational age estimates at birth was essentially the same as the difference at the time of ultrasound scan—2.8 days (with a range of −37 to 38 days). The distributions of LMP- and CRL-based gestational ages appeared similar at the time of ultrasound visit and birth (Fig. 1). The LMP distribution was somewhat flatter at birth, with the most notable divergence between the two distributions at >42 weeks gestation.

**Table 2:** Validity of LMP compared to CRL for gestational age estimation, overall and by parity, rural Bangladesh, 2008–2009

	All	Nulliparous	Parous
Ultrasound visit	(*n* = 353)	(*n* = 134)	(*n* = 219)
Gestational age^a^			
Last menstrual period, days	76.1 (12.5)	78.8 (12.8)	74.4 (12.1)
Crown-rump length, days	73.3 (12.9)	74.6 (12.7)	72.5 (13.0)
LMP-CRL, days	2.8 (10.8)	4.2 (10.0)	1.9 (11.2)
Convergent validity^b^			
Pearson’s correlation coefficient	0.63	0.69	0.61
Bias Correction Factor	0.98	0.95	0.99
Lin’s concordance correlation coefficient (95 % CI)	0.63 (0.56, 0.69)	0.66 (0.56, 0.75)	0.60 (0.52, 0.68)
Live birth	(*n* = 332)	(*n* = 127)	(*n* = 205)
Gestational age^a^			
Last menstrual period, days	276.2 (18.1)	275.9 (17.7)	276.5 (18.4)
Crown-rump length, days	273.4 (14.2)	271.6 (13.6)	274.6 (14.5)
LMP-CRL, days	2.8 (10.7)	4.3 (10.0)	1.9 (11.0)
Convergent validity^b^			
Pearson’s correlation coefficient	0.81	0.83	0.80
Bias correction factor	0.96	0.93	0.97
Lin’s concordance correlation coefficient (95 % CI)	0.77 (0.73, 0.81)	0.77 (0.71, 0.83)	0.77 (0.72, 0.83)
Validity of classifying preterm			
Prevalence of preterm by LMP, % (*n*)	10.8 (36)	13.4 (17)	9.3 (19)
Prevalence of preterm by CRL, % (*n*)	8.7 (29)	11.0 (14)	7.3 (15)
Sensitivity, %	86.2	92.9	80.0
Specificity, %	96.4	96.5	96.3
Positive predictive value, %	69.4	76.5	63.1
Kappa	0.74	0.82	0.68
Validity of classifying post-term			
Prevalence of post-term by LMP, % (*n*)	14.2 (47)	15.0 (19)	13.7 (28)
Prevalence of post-term by CRL, % (*n*)	3.6 (12)	1.6 (2)	4.9 (10)
Sensitivity, %	66.7	87.2	60.0
Specificity, %	88.8	87.4	89.7
Positive predictive value, %	18.2	11.1	23.1
Kappa	0.24	0.18	0.28

Preterm is <259 days, term is 259-293 days, and post-term is >293 days gestational age by ultrasound; the Hadlock equation is used for CRL estimate of gestational age

*LMP* first day of the last menstrual period, *CRL* crown-rump length

^a^Data presented as mean (SD)

^b^In validation analysis: Pearson’s coefficient is a measure of precision; the bias correction factor is a measure of accuracy; and Lin’s concordance correlation coefficient is a measure of reproducibility (accounting for precision and accuracy in the same estimate). Perfect correlation/concordance for each measure = 1

**Fig. 2:**
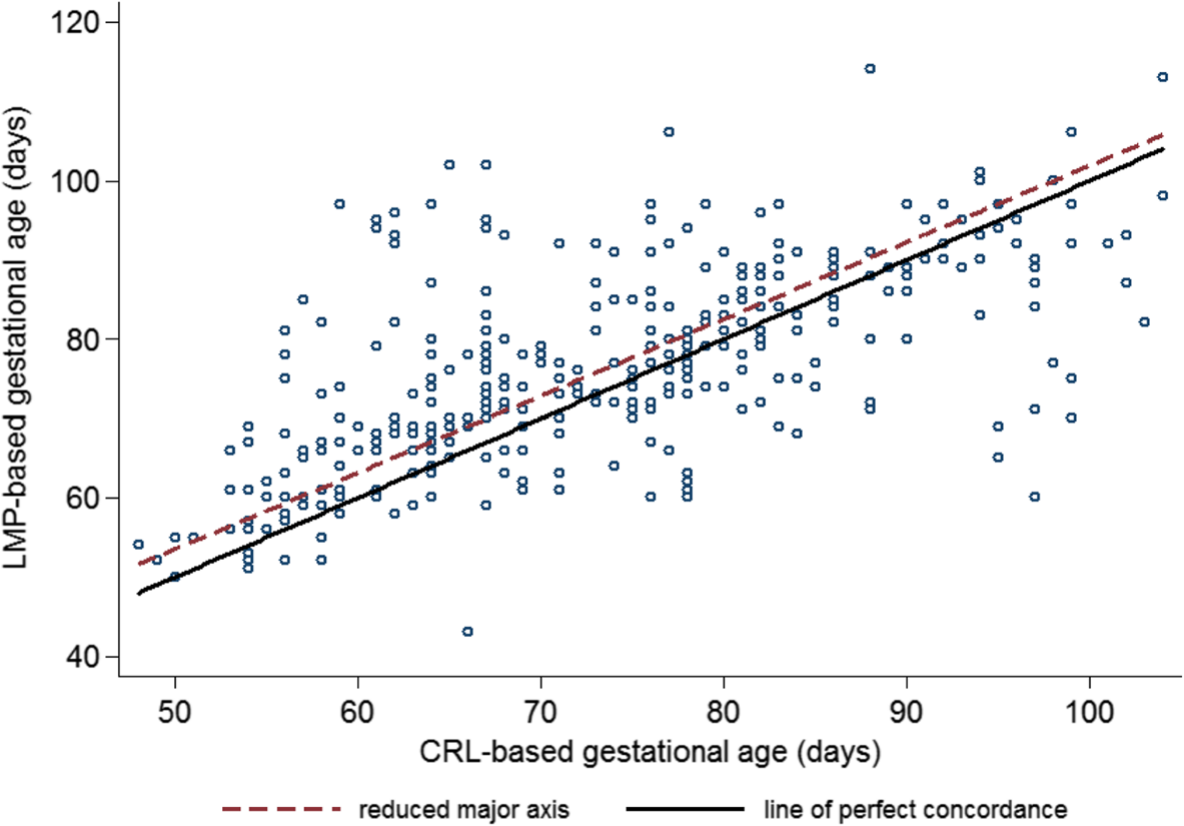
Lin’s concordance correlation plot of last menstrual period (LMP)- vs. crown-rump length (CRL)-based gestational age, *n* = 353

Using validity measures, gestational age by LMP was in good agreement with CRL at the ultrasound visit, with a Pearson’s correlation of 0.63, bias correction factor of 0.98, and Lin’s concordance correlation of 0.63 (Table 2; perfect agreement for each measure = 1). Validity measures in nulliparous and parous women appeared similar, although consistently better among nulliparous than parous women at the time of ultrasound. Validity measures of LMP among live births were also good with a Pearson’s correlation of 0.81, bias correction factor of 0.96, and Lin’s concordance correlation of 0.77. Validity measures at birth appeared similar by parity. Lin’s concordance correlation plot visually shows most estimates relatively close to the line of perfect concordance with deviations both above and below (Fig. 2). For validating term classification, we found sensitivity, specificity, and PPV of LMP to be high for preterm but lower for post-term births (Table 2). Results for the validity of preterm and post-term also appeared similar after stratifying by parity but consistently better for preterm in nulliparous women. We ran the same validity measures using the Intergrowth 21st equation for estimating gestational age with CRL, and the results were similar to those using the Hadlock (Additional file 1: Table S1). The mean (SD) difference in gestational age between LMP and CRL (Intergrowth 21st equation) at the ultrasound visit was 2.5 (10.6) days (*n* = 333) and at live birth was 2.5 (10.5) days (*n* = 315).

We examined the difference in gestational age estimates (LMP-CRL) by maternal and infant characteristics and observed a range of differences from −1.6 to 4.3 days across characteristics (Table 3). Differences between the two gestational age estimates varied by maternal education (higher schooling was associated with a lower difference) and term status (preterm birth was associated with a lower difference). Preterm and post-term births had LMP-CRL differences of a similar magnitude but in opposite directions (−1.57 vs. 1.58 days). We found a large contrast in the differences in estimates by maternal age (4.25 days for women ≤20 years vs. 1.96 days for women >20 years); the difference had a wide confidence interval that included the null.

**Table 3:** Differences in LMP and CRL estimates of gestational age at birth by maternal and infant characteristics, rural Bangladesh, 2008–2009^a^

		Gestational age difference (LMP − CRL), days	
	Number	Difference Mean (SD)	Difference of the difference (95 % CI)^b^
Years of school			
≤6 years	168	4.22 (11.1)	Reference
>6 years	164	1.36 (10.01)	−2.86 (−5.15, −0.58)
Age			
≤20 years	123	4.25 (9.77)	Reference
>20 years	208	1.96 (11.13)	−2.28 (−4.66, 0.10)
Early pregnancy BMI			
Underweight (<18.5 kg/m^2^)	132	2.47 (10.25)	Reference
Normal (≥18.5 kg/m^2^)	200	3.03 (10.97)	0.56 (−1.79, 2.92)
Infant sex			
Male	176	2.94 (10.77)	Reference
Female	156	2.66 (10.60)	−0.29 (−2.60, 2.02)
Term status^c^			
Term	291	3.29 (10.72)	Reference
Preterm	29	-1.57 (10.52)	−4.87 (−8.93, −0.80)
Post-term	12	1.58 (7.66)	−1.71 (−7.86, 4.44)
SGA^d^			
Non-SGA	170	2.38 (11.73)	Reference
SGA	119	2.96 (9.46)	0.58 (−1.97, 3.13)

The Hadlock equation is used for CRL estimate of gestational age

*LMP* first day of the last menstrual period, *CRL* crown-rump length, *SGA* small-for-gestational age by Intergrowth 21^st^ Growth Standard [17], *BMI* body mass index

^a^Live births only

^b^Multivariable linear regression model including all variables in the table

^c^Preterm is <259 days, term is 259–293 days, and post-term is >293 days gestational age by CRL

^d^ *n* = 288 (with newborn weight measured at birth)

## Discussion

Overall, we found that LMP-estimated gestational age was in good agreement with CRL-based estimation, differing by 2.8 days using the Hadlock equation and 2.5 days using the Intergrowth 21st equation. As well, measures of validity showed relatively good agreement between LMP and CRL, with correlation coefficients 0.8 or higher for live births. Further, LMP had high sensitivity and specificity for classifying preterm birth. We found differences in reliability of LMP by years of maternal education and preterm classification, with less educated women and women delivering preterm having a larger discrepancy between their reported LMP and CRL gestational age estimates.

This study validates the use of prospectively collected LMP for estimating gestational age in a low-income, rural setting in Bangladesh, with preterm birth and SGA rates among the highest globally [20]. Uniquely, we used our community-based study to validate this LMP method with early CRL in a reproductive-age population in a rural area. Most LMP validation studies have been done in clinic settings. In our large field study, we collected LMP every month by prospectively tracking married women of reproductive age and using Bangla calendars in the home and urine-testing to identify new pregnancies. This method was likely a major contributor to the good agreement we found between LMP- and CRL-based gestational ages. As such, our findings may be generalizable to studies with similar rigorous methods but may not apply to other settings, e.g., where women report LMP at a first prenatal visit. A limitation was that our planned validation study, which aimed to include 500 pregnancies, had a high attrition rate largely related to fetal loss but also other reasons, such that our final sample size for singleton live births was only 332. In addition, we excluded women who did not enroll before 15 weeks of gestation. This resulted in the women in our validation substudy being different from the overall parent trial, with a preterm birth rate almost half that seen in the larger study, when estimated with LMP. When we examined all live births with LMP dates in the substudy (including those without CRL; *n* = 402), the preterm birth rate was closer to that seen in the parent trial at 13.9 %. Other differences such as higher education, etc., in this validation group may also explain the difference between preterm birth rates with the larger study.

Overall, the characteristics of women in the substudy were similar to the whole trial. Exceptions include that literacy was higher and households having electricity was lower in the substudy compared to the parent study. We were unable to explore all the potential reasons for this paradox; however, availability of schools does not necessarily correlate with availability of electricity to households, and a mother’s education would have occurred while at her parent’s household and not her (current) husband’s household. A final concern was that the sensitivity and specificity for post-term births was found to be somewhat low, and its prevalence was highly inflated when using LMP to estimate gestational age. It is not clear why this occurred, other than the difficulty of predicting a very narrow classification range (only 2 weeks, compared to >12 weeks for preterm classification).

The goal of this research was to investigate the use of LMP as an accurate estimate of gestational age in large field trials such as ours, especially using rigorously collected menstrual dates in a free-living populations, and with the use of a calendar as an aid. Although conducted in a small sample, we also found that the use of a durable, portable, battery-operated ultrasound machine was feasible in a rural setting with limited access to electricity. There was wide acceptance of the measurements by the community, and the study participants considered the procedure safe.

Our sensitivity and specificity estimates of LMP were similar to or better than those reported in two US studies and one in Brazil. In a state-wide study of births in California, Dietz et al. reported a sensitivity of 64 % and a positive predictive value of 59 % for classifying preterm using LMP compared to ultrasound [7]. Similar to our study, the California study found that LMP estimated a slightly higher preterm rate (8.7 vs. 7.9 %) and a much higher post-term rate (10.1 vs. 1.1 %) compared to ultrasound and additionally, that less educated women had greater differences in estimates from the two methods. In a multicenter US study, Hoffman et al. also reported that LMP vs. ultrasound gestational age was longer (by 0.8 days) and classified more births as post-term (4.0 vs. 0.7 %) [6]. In a low-income population in Brazil, LMP compared to ultrasound (at 7–20 weeks gestation) greatly overestimated the rate of both preterm (17 vs. 13 %) and post-term (9.3 vs. 1.3 %) in women with a “sure” LMP [21]. This study used interview-collected LMP at the time of enrollment, which could explain the difference from our study. Other studies validated LMP in specific populations, such as very preterm [8], and may not be comparable to the current study.

First trimester CRL, as used in this study, is considered the best measurement for gestational age determination [10], as it is both accurate and precise, predicting 94 % of delivery dates within 14 days [22]. Other research also shows early pregnancy CRL to be the best at estimating gestational age among ultrasound measures of fetal size [22, 23], presumably because measurements at this time should be before any discernable growth restriction that could impact gestational age estimation. In our findings, the difference between LMP and CRL estimates were similar for SGA and non-SGA pregnancies. Thus, it is not apparent that fetal growth restriction impacted our ultrasound measurements; however, this was not a specific aim of the study to investigate.

We expected that measurements could also be different based on maternal characteristics, especially those that could impact a woman’s record of LMP. We found that LMPs reported by better educated women had better agreement with CRL, which agrees with another US study [6]. Perhaps in our study, the discrepancy was due to the woman’s ability to read and mark the calendar we provided. We also found that for women who delivered preterm, there was a larger difference between the LMP and CRL estimates. It is unclear why preterm would predict a larger difference between gestational age estimates than term, although the gestational age range for preterm births is much wider (24 to <37 weeks) than the narrower range defining term birth (37–42 weeks) and post-term birth (42 to <44 weeks) and more of these values are farther from the expected normal length of gestation. We did not observe a difference in LMP and CRL estimates by maternal BMI. Although ultrasound measurements can be impacted by maternal obesity [24], rates of overweight were very low in this population; there were only seven overweight women in our study (2 %). Finally, we observed comparable validity results for nulliparous and parous women, similar to a previous study [21].

## Conclusions

Our community-based, prospectively collected LMP was a valid measure for estimating gestational age and preterm rates in a low-income, low-education, rural setting. This result is important as our parent trial found a significant reduction in the LMP-based preterm birth rate with multiple micronutrient supplementation [11]. As well, a major public health concern in the developing world is reduction of preterm births [1], and the use of this method could provide valid estimates of preterm births for monitoring progress toward this goal. Although ultrasound is clearly preferred and increasingly available, we expect that LMP collected prospectively will allow public health researchers to test the impact of pregnancy interventions on preterm birth and validly estimate gestational age in rural settings.

## Additional file

Additional file 1: Table S1. Validity of LMP compared to CRL (using the Intergrowth 21st equation [19]) for gestational age estimation, overall and by parity, rural Bangladesh, 2008–2009. (DOCX 16 kb)

## Abbreviations

CRL: Crown-rump length
LMIC: Low-income and middle-income countries
LMP: Last menstrual period
SGA: Small-for-gestational age
