# Supplementary material for: A home calendar and recall method of last menstrual period for estimating gestational age in rural Bangladesh: a validation study
Source: J Health Popul Nutr. 2016 Oct 21;35:34. doi: 10.1186/s41043-016-0072-y (PMC5073953; doi:10.1186/s41043-016-0072-y)
Supplement: Additional file 1: Table S1. — Validity of LMP compared to CRL (using the Intergrowth 21st equation [19]) for gestational age estimation, overall and by parity, rural Bangladesh, 2008–2009. (DOCX 16 kb) [file 41043_2016_72_MOESM1_ESM.docx]

**Additional file 1: Table S1.** Validity of LMP compared to CRL (using the Intergrowth 21^st^ equation[[19](#_ENREF_19)]) for gestational age estimation, overall and by parity, rural Bangladesh, 2008-2009

|  | All | Nulliparous | Parous |
| --- | --- | --- | --- |
| Ultrasound visit | (*n*=333) | (*n*=129) | (*n*=204) |
| Gestational age^1^ |  |  |  |
| Last menstrual period, days | 77.2 (11.9) | 79.6 (12.3) | 75.7 (11.4) |
| Crown-rump length, days | 74.7 (11.3) | 75.4 (11.3) | 74.3 (11.3) |
| LMP-CRL, days | 2.5 (10.6) | 4.1 (9.7) | 1.4 (11.1) |
|  |  |  |  |
| Convergent validity^2^ |  |  |  |
| Pearson’s correlation coefficient | 0.58 | 0.67 | 0.52 |
| Bias Correction Factor | 0.98 | 0.94 | 0.99 |
| Lin’s concordance correlation coefficient (95 % CI) | 0.57  (0.49, 0.64) | 0.62  (0.52, 0.72) | 0.52  (0.42, 0.62) |
|  |  |  |  |
| Live Birth | (*n*=315) | (*n*=122) | (*n*=193) |
| Gestational age^1^ |  |  |  |
| Last menstrual period, days | 276.3 (18.3) | 275.9 (17.8) | 276.6 (18.7) |
| Crown-rump length, days | 273.9 (14.3) | 271.7 (13.5) | 275.2 (14.6) |
| LMP-CRL, days | 2.5 (10.5) | 4.2 (9.7) | 1.4 (10.8) |
|  |  |  |  |
| Convergent validity^2^ |  |  |  |
| Pearson’s correlation coefficient | 0.82 | 0.84 | 0.82 |
| Bias Correction Factor | 0.96 | 0.93 | 0.97 |
| Lin’s concordance correlation coefficient (95 % CI) | 0.79  (0.75, 0.83) | 0.78  (0.72, 0.84) | 0.79  (0.74, 0.84) |
|  |  |  |  |
| Validity of classifying preterm |  |  |  |
| Prevalence of preterm by LMP, % (*n*) | 10.8 (34) | 13.1 (16) | 9.3 (18) |
| Prevalence of preterm by CRL, % (*n*) | 9.5 (30) | 13.1 (16) | 7.3 (14) |
| Sensitivity, % | 83.3 | 81.3 | 85.7 |
| Specificity, % | 96.9 | 97.2 | 96.7 |
| Positive predictive value, % | 73.5 | 81.3 | 66.7 |
| Kappa | 0.76 | 0.78 | 0.73 |
|  |  |  |  |
| Validity of classifying post-term |  |  |  |
| Prevalence of post-term by LMP, % (*n*) | 13.7 (43) | 13.9 (17) | 13.5 (26) |
| Prevalence of post-term by CRL, % (*n*) | 4.1 (13) | 1.6 (2) | 5.7 (11) |
| Sensitivity, % | 61.5 | 100.0 | 54.5 |
| Specificity, % | 88.4 | 87.5 | 89.1 |
| Positive predictive value, % | 18.6 | 11.8 | 23.1 |
| Kappa | 0.24 | 0.19 | 0.27 |

LMP, first day of last menstrual period; CRL, crown-rump length; Preterm is <259 days, term is 259-293 days, and post-term is >293 days gestational age by ultrasound.

^1^Data presented as mean (SD).

^2^ In validation analysis: Pearson’s coefficient is a measure of precision; the Bias Correction Factor is a measure of accuracy; and Lin’s concordance correlation coefficient is a measure of reproducibility (accounting for precision and accuracy in the same estimate). Perfect correlation/concordance for each measure = 1.
